# Predictive value of the neutrophil percentage-to-albumin ratio for coronary atherosclerosis severity in patients with CKD

**DOI:** 10.1186/s12872-024-03896-x

**Published:** 2024-05-28

**Authors:** Mengqi Zhao, Xin Huang, Yang Zhang, Zuoyan Wang, Songli Zhang, Jianjun Peng

**Affiliations:** Department of Cardiology, Beijing Shijitan Hospital, Capital Medical University, Beijing, 100038 China

**Keywords:** Coronary atherosclerosis, Chronic kidney disease, Gensini score, Neutrophil percentage-to-albumin ratio

## Abstract

**Background:**

The neutrophil percentage-to-albumin ratio (NPAR), which is defined as the percentage of neutrophils divided by the concentration of albumin, is a cost-effective and readily available biomarker of inflammation. This study aimed to evaluate the association between the NPAR and the severity of coronary atherosclerosis in patients with chronic kidney disease (CKD).

**Methods:**

A total of 280 CKD patients who underwent coronary angiography were retrospectively enrolled in this study. The severity of coronary atherosclerosis was evaluated using the Gensini score (GS). Patients were divided into low-, medium- and high-NPAR groups according to the tertiles of the NPAR values. Logistic regression analysis was conducted to analyze the relationship between the NPAR and the GS. The cutoff points for the sensitivity and specificity of the NPAR in predicting the GS were estimated via receiver operating characteristic (ROC) analysis.

**Results:**

There was a higher prevalence of coronary artery disease (CAD) among CKD patients with higher NPARs (*P* =0.041). More patients in the high-NPAR group had complex CAD (triple-vessel disease and/or left main coronary artery stenosis) and chronic total occlusion lesions, and more of these patients required revascularization therapy (P<0.05). Multivariate logistic regression analysis revealed a significant positive correlation between the NPAR and the severity of coronary stenosis (adjusted OR 2.68, 95% CI 1.25-5.76, *p*=0.012), particularly among female and older (age ≥65) patients. The ROC analysis indicated that the optimal cutoff value for the NPAR in predicting severe coronary artery stenosis (GS>60) in CKD patients was 1.91 (sensitivity 0.495, specificity 0.749), with an area under the curve (AUC) of 0.650 (95% CI 0.581-0.719, *P*<0.001). A subgroup analysis according to sex revealed that the NPAR exhibited stronger predictive value in female patients (AUC 0.730, 95% CI 0.643-0.817) than in male patients (AUC 0.565, 95% CI 0.460-0.670) (*P*<0.001), and the optimal cutoff value for the NPAR in female patients was 1.80 (sensitivity 0.667, specificity 0.705).

**Conclusions:**

Our study demonstrated that the NPAR is independently associated with the severity of coronary atherosclerosis in CKD patients, especially in female and elderly patients (≥65 years old). Moreover, the NPAR can effectively predict the severity of coronary atherosclerosis, exhibiting greater predictive value in females than in males.

## Background

Coronary artery disease (CAD) is one of the major causes of mortality worldwide, and its mortality rate has gradually increased to more than 27% [1], particularly in individuals with chronic kidney disease (CKD) [2]. In these patients, the risk of death due to cardiovascular disease is 30 times higher than that in the general population [3]. It is well known that the main cause of CAD is coronary atherosclerosis. Coronary atherosclerotic lesions in patients with CKD are complex and serious, leading to a worse prognosis and greater financial burden for these patients [4]. However, evaluating the severity of coronary atherosclerosis in these patients is difficult; coronary angiography (CAG) and percutaneous coronary intervention are imperative for the diagnosis and treatment of coronary atherosclerosis, and the contrast agents that are used during these procedures are excreted through the kidneys, which might damage renal function and facilitate CKD progression. Thus, efforts have been made to identify alternative noninvasive indices to overcome this problem.

For CKD patients, the high rates of mortality due to cardiovascular disease cannot be adequately explained by traditional risk factors, and the pivotal role of inflammation in the mechanism underlying disease in these patients has been increasingly recognized [5, 6]. Studies have shown that inflammation plays a role in initiating both CAD and CKD. Various markers of inflammatory reactions, including high-sensitivity C-reactive protein, fibrinogen, and interleukin-6 as well as the neutrophil-to-lymphocyte ratio, have been demonstrated to have independent predictive value for coronary atherosclerosis and to be associated with CAD prognosis [7–10]. However, since patients with CKD undergo CAG infrequently and many clinical studies exclude CKD patients, only a limited number of studies have reported that inflammation might also accelerate the progression of coronary atherosclerosis and adverse cardiovascular outcomes in this population [11–13]. More comprehensive investigations are needed to support this theory. Recently, the neutrophil percentage-to-albumin ratio (NPAR), which is an emerging indicator of inflammation, was shown to be associated with the clinical outcome of many diseases, such as nonalcoholic fatty liver disease, advanced liver fibrosis, stroke, and severe CAD [14–16]. However, the most recently conducted studies on this topic did not include patients who also had CKD. Whether a similar relationship between the NPAR and the severity of coronary atherosclerosis is observed in patients with CKD, which constitutes a complex pathological background, remains unclear.

Thus, this study aimed to comprehensively explore the potential correlation between the NPAR and the severity of coronary atherosclerosis in CKD patients and to identify potential new predictors for such patients in clinical practice.

## Methods

### Study population

The study protocol was approved by the Ethics Committee of our institution [No. sjtky11-1x-2023(019)]. All the procedures were conducted following the guidelines and principles of the Declaration of Helsinki, and written informed consent was obtained from all the patients. Our population consisted of CKD patients who underwent nonurgent coronary angiography but did not receive dialysis therapy at Beijing Shijitan Hospital of Capital Medical University, Beijing, China, between January 2021 and January 2023. CKD was considered in patients with a history of renal failure (requiring specialist follow-up and/or specific treatment) or in those with an estimated glomerular filtration rate (eGFR) of less than 60 mL/min/1.73 m^2^, as defined by the Kidney Disease-Epidemiology Collaboration (CKD-EPI) formula, upon admission [17]. The exclusion criteria were as follows: (1) acute myocardial infarction; (2) history of coronary artery bypass grafting or percutaneous coronary intervention; (3) heart disease beyond coronary artery disease, such as myocarditis, valvular diseases, and severe heart failure (left ventricular ejection fraction <30%); (4) severe hepatic dysfunction; (5) ongoing infection or recovering from various acute and chronic infections; (6) thyroid disorders, tuberculosis, inflammatory bowel disease, autoimmune diseases, or hematological disorders; or (7) incomplete clinical data. We obtained medical histories by reviewing the patients’ electronic medical records, and the medical histories included age, sex, ethnicity, history of smoking or alcohol consumption, hypertension, diabetes mellitus (DM), dyslipidemia, history of renal insufficiency and dialysis, systolic and diastolic blood pressure within 1 hour of admission, and diagnosis and treatment during hospitalization. Hypertension was defined as a systolic blood pressure > 140 mmHg and/or diastolic blood pressure > 90 mmHg or the need for antihypertensive medication. The diagnosis of DM was based on a previous history of diabetes (treated or untreated), fasting glycemia > 126 mg/dl, random glycemia > 200 mg/dl or HbA1c > 48 mmol/L. Dyslipidemia was defined as total cholesterol (TC) > 240 mg/dl, serum triglyceride (TG) > 150 mg/dl, high‐density lipoprotein cholesterol (HDL‐C) < 40 mg/dl, low‐density lipoprotein cholesterol (LDL‐C) ≥ 160 mg/dl, or diagnosis/treatment of dyslipidemia.

### Laboratory measurements

Before CAG, the levels of white blood cells (WBCs), neutrophils, hemoglobin (Hb), platelets, albumin, blood urea nitrogen (BUN), serum creatinine (SCr), eGFR, uric acid (UA), TC, TG, phosphorus, potassium, HDL-C, LDL-C, corrected calcium, fibrinogen, HbA1c, thyroid-stimulating hormone (TSH), free triiodothyronine (FT3), and free thyroxine (FT4) were measured after 12 h of overnight fasting. Venous blood samples were obtained from the antecubital vein of all the participants. The blood parameters were determined by the clinical laboratory of the Beijing Shijitan Hospital of Capital Medical University. The NPAR was calculated after measuring the neutrophil percentage and albumin levels in the same blood samples that were collected at admission.

### CAG

CAG was performed by at least two experienced clinicians using an X-ray system according to the standard Judkins technique, and all the patients provided informed consent. CAD was defined as at least 50% stenosis of the vessel lumen diameter in one of the main coronary arteries (the right coronary artery, the left circumflex coronary artery, or the left anterior descending coronary artery). The severity of coronary atherosclerosis was evaluated using the Gensini score (GS) [18]. The GS was calculated as follows. Points were assigned according to the percentage of luminal narrowing: 1 point for 1%-25% occlusion, 2 points for 26%-50%, 4 points for 51%-75%, 8 points for 76%-90%, 16 points for 91%-99%, and 32 points for complete occlusion. Each of these point values was multiplied by the corresponding factors accounting for the location of the obstruction. Finally, the total GS for each patient is expressed as the sum total score of each lesion [19].

Diseased coronary vessels were categorized as single-, double-, or triple‐vessel stenosis based on the number of stenotic vessels among the left anterior descending artery, left circumflex artery, and right coronary artery. Patients with ≥ 50% stenosis in the left main coronary artery diameter were diagnosed with left main coronary artery stenosis (LMCA). Complex CAD was defined as triple-vessel disease (TVD) and/or LMCA. Chronic total occlusion (CTO) was diagnosed in patients with arteries with atherosclerotic luminal narrowing resulting in TIMI grade 0 flow for more than 3 months based on angiographic findings or the duration of patient symptoms and clinical presentation [20].

### Statistical analysis

SPSS 26.0 software (IBM Corporation, Armonk, NY, USA) was used for the statistical analyses. Normally distributed measurements are expressed as the mean ± SD. Comparisons between two groups were made with independent-samples t tests, and comparisons among multiple groups were made using one-way analysis of variance. Nonnormally distributed measurements are expressed as the median (IQR). Comparisons between two groups were made using the Mann‒Whitney U test, and comparisons among multiple groups were made using the Kruskal‒Wallis H test. Count data are expressed as the number of patients (%), and the χ2 test was used for comparisons between groups. Spearman analysis was used for correlation analysis. Univariate and multivariate logistic regression analyses were performed to evaluate the factors affecting coronary atherosclerosis severity. The best cutoff values for the plasma NPAR were determined by receiver operating characteristic (ROC) analysis and area under the curve (AUC) calculation. A *P* value less than 0.05 was considered to indicate statistical significance.

## Results

### Clinical characteristics of the participants

A total of 280 CKD patients who underwent CAG were classified into three groups according to the tertiles of the NPAR: patients with NPAR ≤ 16.30 were included in the low-NPAR group (*n* = 93), patients with 16.30 < NPAR ≤ 19.12 were included in the medium-NPAR group (*n*=94), and patient with NPAR>19.12 were included in the high-NPAR group (*n*= 93 patients). The baseline patient characteristics and blood test results of the three groups are presented in detail in Table 1. The average age of all the patients was 74.02 years; there were 133 (47.50%) male patients and 147 (52.50%) female patients. The mean eGFR was 46.01 mL/min/1.73 m^2^.

**Table 1:** Baseline clinical characteristics

Variables	low-NPAR group (*n*=93)	medium-NPAR group (*n*=94)	high-NPAR group (*n*=93)	*P* value
Age (years)	71.92±9.35	75.73±11.08	74.38±11.26	**0.047**
Male, n (%)	35 (37.63)	47 (50.00)	51 (54.84)	0.053
Han ethnicity, n (%)	92 (98.92)	89 (94.68)	89 (95.70)	0.264
SBP (mmHg)	137.38±21.32	140.18±22.88	137.28±25.66	0.628
DBP (mmHg)	79.14±10.13	78.98±13.73	75.86±16.20	0.180
Comorbidities and medical history, n (%)				
Smoking, n (%)	21 (22.58)	30 (31.91)	36 (38.71)	0.058
Alcohol intake, n (%)	10 (10.75)	16 (17.02)	18 (19.35)	0.249
Hypertension, n (%)	79 (84.95)	84 (89.36)	80 (86.02)	0.649
Hypertension grade				0.645
Grade 1	8 (8.60)	9 (9.57)	8 (8.60)	
Grade 2	25 (26.88)	25 (26.60)	16 (17.20)	
Grade 3	46 (49.46)	50 (53.19)	56 (60.22)	
DM, n (%)	44 (47.31)	43 (45.74)	57 (61.29)	0.065
Dyslipidemia, n (%)	87 (93.55)	87 (92.55)	87 (93.55)	0.952
Laboratory parameters				
WBC (10^9^/L)	6.58±1.84	6.97±2.02	9.06±3.08	**<0.001**
Neutrophil (10^9^/L)	3.98±1.32	4.83±1.53	6.94±2.70	**<0.001**
NPAR	1.45±0.15	1.77±0.08	2.17±0.25	**<0.001**
Hemoglobin (g/dL)	129.45±16.97	124.23±19.87	119.26±20.25	**0.002**
Platelet (10^9^/L)	213.72±52.21	218.07±75.58	234.78±87.73	0.120
Albumin (g/dL)	41.57±3.16	38.89±2.91	35.14±4.01	**<0.001**
BUN (mmol/L)	8.46±2.41	8.91±2.54	10.90±3.67	**<0.001**
SCr (μmol/L)	112.34±36.73	119.04±30.95	137.44±48.92	**<0.001**
eGFR*	49.47±9.77	46.26±9.68	42.31±12.02	**<0.001**
UA (μmol/L)	408.80±112.26	427.40±112.42	449.31±126.80	0.064
TC (mmol/L)	4.38±1.05	4.18±1.12	4.21±1.38	0.488
TG (mmol/L)	2.00±1.27	1.68±0.93	1.73±0.98	0.094
Phosphorus (mmol/L)	1.19±0.20	1.17±0.17	1.22±0.27	0.284
Potassium (mmol/L)	4.25±0.36	4.20±0.36	4.21±0.39	0.607
HDL-C (mmol/L)	1.10±0.25	1.02±0.21	0.96±0.25	**0.001**
LDL-C (mg/dL)	2.79±0.91	2.70±0.93	2.78±1.03	0.797
Corrected calcium (mmol/L)	2.38±0.13	2.31±0.10	2.22±0.15	0.086
Fibrinogen (g/L)	3.09±0.52	3.28±0.60	3.95±1.29	**<0.001**
HbA1c (%)	6.67±1.18	6.71±1.15	7.32±1.56	**0.001**
TSH (uIU/mL)	4.61±13.03	3.56±8.60	2.87±2.75	0.428
FT3 (pg/ml)	2.57±0.41	2.48±0.53	2.29±0.40	**<0.001**
FT4 (ng/dl)	1.25±0.26	1.30±0.31	1.26±0.21	0.389
Coronary procedural information				
CAD	75 (80.65)	84 (89.36)	86 (92.47)	**0.041**
Gensini score	37.11±38.52	41.71±38.67	57.99±37.53	**0.001**
Single-vessel disease	20 (21.51)	28 (29.79)	12 (12.90)	**0.019**
Double-vessel disease	24 (25.81)	19 (20.21)	22 (23.66)	0.658
Triple-vessel disease	31 (33.33)	37 (39.36)	52 (55.91)	**0.006**
LMCA	3 (3.23)	7 (7.45)	10 (10.75)	0.136
CTO	9 (9.68)	13 (13.83)	26 (27.96)	**0.002**
PCI	56 (60.22)	65 (69.15)	75 (80.65)	**0.010**

The data are presented as the means ± SDs or n (%). *Calculated using the CKD-EPI formula [17]. *SBP* Systolic blood pressure, *DBP* Diastolic blood pressure, *DM* Diabetes mellitus, *WBC* White blood cell, *NPAR*,Neutrophil percentage-to-albumin ratio, *BUN* Blood urea nitrogen, *SCr* Serum creatinine; *eGFR* Estimated glomerular filtration rate, *UA* Uric acid, *TC* Total cholesterol, *TG* Triglyceride, *HDL-C* High-density lipoprotein cholesterol, *LDL-C* Low-density lipoprotein cholesterol

As shown in Table 1, patients in the high-NPAR group had a higher prevalence of CAD (P =0.041). The mean GSs of the low-, medium- and high-NPAR groups were 37.11 ± 38.52, 41.71 ± 38.67, and 57.99 ± 37.53, respectively, and the correlation between the NPAR and the GS was significant (*p*<0.001). In addition, the high-NPAR group had fewer patients with single-vessel disease, more patients with three-vessel disease (*P* <0.05), a higher percentage of patients with CTO lesions (*P* =0.002), and more patients who required revascularization treatment (*P* =0.010). The prevalence of LMCA lesions did not significantly differ among the three groups. The levels Hb, BUN, SCr, HbA1c, and FT3 were higher while the eGFR and HDL‐C levels were lower in the high-NPAR group than in the other two groups (all *p* < 0.05). No significant between-group differences in sex, ethnicity, blood pressure, history of smoking or alcohol consumption, hypertension, DM, dyslipidemia, platelet count, UA, TC, TG, phosphorus, potassium, LDL-C, corrected calcium, TSH or FT4 were observed among the three NPAR groups.

### Association between the NPAR and the severity of coronary atherosclerosis

**Fig. 1:**
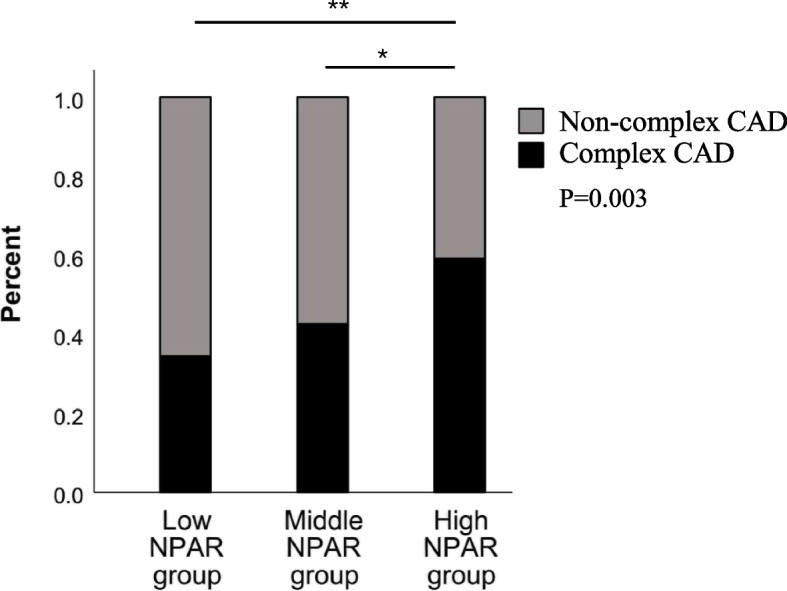
Bar graph showing the relationship between the NPAR and the proportion of patients with CKD who also had complex CAD (TVD and/or LMCA)

As shown in Fig. 1, patients with a higher NPAR had higher rates of complex CAD (TVD and/or LMCA), and the differences among the three groups were significant (*P*=0.003). In addition, by using the GS to assess the severity of coronary artery stenosis, we also found that as the NPAR increased, the GS decreased (*P*=0.001, shown in Table 1).

### Multivariate logistic regression analysis for factors that impact the severity of coronary atherosclerosis stenosis

As shown in Table 2, univariate logistic regression analysis revealed that the following factors were associated with the GS: male sex (crude OR 1.54, 95% CI 1.00-2.38, *p*=0.051), age (crude OR 1.00, 95% CI 0.98-1.02, *p*=0.845), hypertension (crude OR 1.94, 95% CI 1.01-3.71, *p*=0.046), DM status (crude OR 2.55, 95% CI 1.64-3.97, *p*<0.001), the NPAR (crude OR 4.40, 95% CI 2.23-8.67, *p*<0.001), BUN (crude OR 1.16, 95% CI 1.07-1.25, *p*<0.001), SCr (crude OR 1.01, 95% CI 1.00-1.01, *p*=0.007), eGFR (crude OR 0.97, 95% CI 0.95-0.99, *p*=0.006), UA (crude OR 1.00, 95% CI 1.00-1.00, *p*=0.028), HDL-C (crude OR 0.40, 95% CI 0.16-0.99, *p*=0.046) and HbA1c (crude OR 1.30, 95% CI 1.09-1.53, *p*=0.003). These factors were subsequently included in a multivariate logistic regression analysis. The results showed that male sex (adjusted OR 1.99, 95% CI 1.09-3.62; *p*=0.025), DM (adjusted OR 1.88, 95% CI 1.07-3.28, *p*=0.027) and the NPAR (adjusted OR 2.68, 95% CI 1.25-5.76, *p*=0.012) were independent risk factors for elevated GSs.

**Table 2:** Univariate and multivariate logistic regression analyses for variables associated with the GS

Factor	Univariate analysis		Multivariate analysis	
	OR (95% CI)	*P* value	OR (95% CI)	*P* value
Male, n (%)	1.54 (1.00-2.38)	0.051	1.99 (1.09-3.62)	**0.025**
Age (years)	1.00 (0.98-1.02)	0.845	1.00 (0.97-1.03)	0.983
Hypertension, n (%)	1.94 (1.01-3.71)	**0.046**	1.56 (0.78-3.11)	0.210
DM, n (%)	2.55 (1.64-3.97)	**<0.001**	1.88 (1.07-3.28)	**0.027**
Dyslipidemia, n (%)	1.65 (0.69-3.94)	0.256	1.28 (0.52-3.18)	0.589
NPAR	4.40 (2.23-8.67)	**<0.001**	2.68 (1.25-5.76)	**0.012**
Hemoglobin (g/dL)	0.99 (0.98-1.00)	0.135		
BUN (mmol/L)	1.16 (1.07-1.25)	**<0.001**	1.08 (0.97-1.19)	0.143
SCr (μmol/L)	1.01 (1.00-1.01)	**0.007**	0.99 (0.98-1.00)	0.151
eGFR	0.97 (0.95-0.99)	**0.006**	0.97 (0.92-1.02)	0.193
UA (μmol/L)	1.00 (1.00-1.00)	**0.028**	1.00 (1.00-1.00)	0.478
HDL-C (mmol/L)	0.40 (0.16-0.99)	**0.046**	0.86 (0.32-2.34)	0.772
LDL-C (mg/dL)	1.04 (0.83-1.31)	0.705		
HbA1c (%)	1.30 (1.09-1.53)	**0.003**	1.06 (0.86-1.31)	0.585

Adjusted for sex, age, hypertension, DM, dyslipidemia, platelet count, BUN, SCr, eGFR, UA, HDL-C and HbA1c. *95% CI* 95% confidence interval, *OR* Odds ratio, *DM* Diabetes mellitus, *BUN* Blood urea nitrogen, *NPAR* Neutrophil percentage-to-albumin ratio, *BUN* Blood urea nitrogen, *SCr* Serum creatinine, *eGFR* Estimated glomerular filtration rate, *UA* Uric acid, *HDL-C* High-density lipoprotein cholesterol, *LDL-C* Low-density lipoprotein cholesterol

Patients were further categorized based on age (<65 or ≥65 years), sex, and history of hypertension, DM, or dyslipidemia. As shown in Table 3, univariate logistic regression analysis revealed a noteworthy and positive correlation between the NPAR and the GS in the older subgroup (OR=5.05, 95% CI 2.34-10.86, *P*<0.001) as well as in the female subgroup (OR=7.21, 95% CI 2.74-18.99, *P*<0.001). These correlations persisted after adjusting for confounding variables. According to the multivariate analysis, the NPAR was also significantly associated with the GS in patients who also had DM (OR=4.25, 95% CI 1.50-12.02; *P*=0.006).

**Table 3:** Logistic regression analysis to assess the correlation between the NPAR and the GS in different subgroups

Factor	Univariate analysis		Multivariate analysis	
	OR (95% CI)	*P* value	OR (95% CI)	*P* value
**Sex**				
Male	2.17(0.80-5.85)	0.126	1.75(0.59-5.17)	0.310
Female	7.21(2.74-18.99)	**<0.001**	4.02(1.33-12.14)	**0.014**
**Age**				
<65	2.42(0.55-10.64)	0.241	2.30(0.29-18.04)	0.428
≥65	5.05(2.34-10.86)	**<0.001**	4.28(1.82-10.07)	**0.001**
**DM**				
Yes	4.00(1.59-10.02)	**0.003**	4.25(1.50-12.02)	**0.006**
No	3.52(1.24-9.93)	**0.018**	1.99(0.63-6.33)	0.241

Adjusted for age, sex, DM, hypertension, dyslipidemia, eGFR, SCr, HbA1c, and HDL-C. *95% CI* 95% confidence interval, *OR* odds ratio, *DM* Diabetes mellitus

### The diagnostic value of the NPAR for the severity of coronary atherosclerosis

**Fig. 2:**
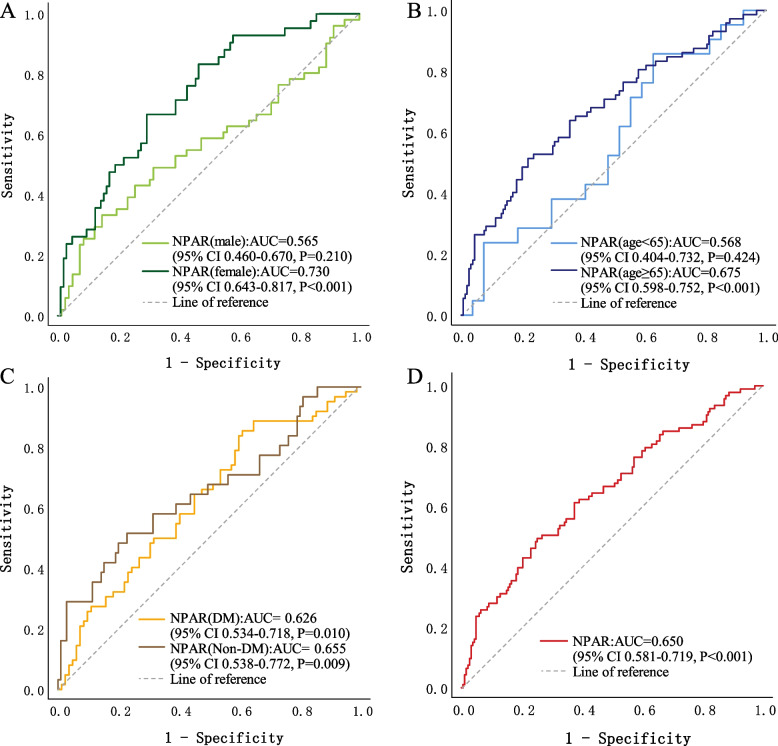
Comparison of receiver operating characteristic (ROC) curves for different populations. These findings show the ability of the NPAR to predict severe coronary stenosis. The AUC indicates the area under the curve

Using ROC curves, we compared the ability of the NPAR to predict severe coronary artery stenosis (GS>60) in different patient groups. As shown in Fig. 2A, the AUC for the NPAR in female patients was 0.730 (95% CI 0.643-0.817, *P*<0.001), while that in male patients was 0.565 (95% CI 0.460-0.670, *P*=0.210) and had no predictive value. In female patients, the best cutoff value for the NPAR was 1.80 (sensitivity of 0.667, specificity of 0.705). As shown in Fig. 2B, for patients aged ≥65 years, the AUC for the NPAR was 0.675 (95% CI 0.598-0.752, *P*<0.001). However, for patients aged <65 years, the NPAR had no predictive value (AUC 0.568, 95% CI 0.404-0.732; *P*=0.424). As shown in Fig. 2C, the NPAR demonstrated predictive value in both patients with diabetes and patients without diabetes. The AUC for the NPAR in patients with diabetes was 0.626 (95% CI 0.534-0.718, *P*=0.010), while in patients without diabetes, it was 0.655 (95% CI 0.538-0.772, *P*=0.009). As shown in Fig. 2D, among all the included patients, the NPAR had an AUC of 0.650 (95% CI 0.581-0.719, *P*<0.001). The optimal cutoff value of the NPAR was 1.91 (sensitivity 0.495, specificity 0.749).

## Discussion

This study revealed that in the population of patients with CKD, the NPAR is independently associated with the severity of coronary atherosclerosis. This association is especially pronounced in female and elderly patients (aged ≥ 65 years). Furthermore, the NPAR exhibited a good ability to predict the severity of coronary stenosis.

Inflammation is the initiating factor for both atherosclerosis and CKD. The NPAR, which combines two parameters that are classically used for clinical evaluation, represents a novel biomarker that reflects systemic inflammation. The formation and progression of atherosclerotic plaques involve a complex inflammatory cascade [21, 22]. Neutrophils promote inflammatory responses and oxidative stress [23] and play pivotal roles in processes such as plaque instability, rupture, and thrombus formation [24, 25]. Previous studies have indicated a positive correlation between neutrophils and the GS in CAD patients [26, 27]. Additionally, Avanzas et al. have shown that neutrophils can serve as independent predictors for the presence of multiple complex stenoses [28]. Albumin, which is known as a negative acute-phase reactant [29], suppresses oxidative stress and inflammatory responses [30, 31]. Hypoalbuminemia may increase blood viscosity and impair endothelial function, thereby accelerating the progression of atherosclerosis [32]. Several studies have provided compelling evidence that lower serum albumin concentrations promote the development and progression of coronary atherosclerosis and are associated with poor clinical outcomes in CAD patients [31, 33, 34].

CKD is also characterized by a chronic inflammatory state [35]. Neutrophils can mediate inflammation in renal injury through various biochemical mechanisms, leading to further tissue damage and impaired renal function [36]. In CKD patients, the loss of protein and reduced intake can lead to hypoalbuminemia. Don et al. reported that hypoalbuminemia serves as a robust predictive indicator for both the incidence and mortality of CKD patients [29]. Thus, previous studies have indicated a negative association between the eGFR and neutrophil count and a positive relationship between the eGFR and albumin levels [37, 38]. Consistent with these studies, our research also revealed a statistically significant decrease in the eGFR with increasing NPAR in predialysis patients with stage 3-5 CKD.

Based on the evidence presented, we hypothesized and demonstrated that CKD patients with elevated NPARs have a higher prevalence of CAD. More patients in the high-NPAR group had complex CAD and chronic total occlusion lesions, and more patients in this group required revascularization therapy. More importantly, a higher NPAR could be considered an independent risk factor for coronary atherosclerosis (adjusted OR 2.68, 95% CI 1.25-5.76; *p*=0.012). ROC analysis revealed that the AUC of the NPAR for predicting the severity of coronary artery stenosis was 0.650 (95% CI 0.581-0.719, *P*<0.001). Recent studies have also shown that the NPAR exhibits predictive value for the prognosis of cardiovascular events, including acute myocardial infarction, heart failure, and cardiogenic shock [16, 39–41].

Furthermore, among women with CKD, the NPAR is more strongly correlated with the GS than it is in men, and the NPAR has the highest predictive value for the severity of coronary artery lesions. While females are commonly known to have a longer life expectancy than males, in part due to lower incidence rates of cardiovascular disease, this trend might not hold for patients who also have CKD [42]. Research has indicated that CKD is more prevalent in females (7.7% in females versus 5.6% in males) [43]. Compared with males, females also exhibit greater renal vascular resistance, lower GFRs, and reduced renal blood flow [42]. Current research suggests that renal insufficiency is one of the risk factors for coronary atherosclerosis [44]. In addition, CAD in female patients is often characterized by atypical symptoms, microvascular involvement, and plaque formation or progression, which makes the diagnosis of CAD in females challenging [45]. These sex disparities may be driven by the effects of hormonal variation, particular hormonal variation from pre- to postmenopausal stages, on regulating inflammation. For example, serum estradiol (E2) regulates G protein-coupled estrogen receptors, reducing the production of inflammatory factors, such as IL-6 and TNF-α, and thereby significantly lowering the body's inflammatory response; this has been shown to inhibit the progression of atherosclerosis [46]. After menopause, the combined effect of complex CKD in females and decreased endogenous E2 secretion might more significantly increase the risk of coronary atherosclerosis in females than in males. Other evidence from extensive studies on female cohorts also suggested that cardiovascular events are associated with increased levels of inflammatory mediators, particularly among postmenopausal women [47]. All the women who were included in our study were menopausal; thus, ours is the first study to report a more significant correlation between the NPAR and coronary atherosclerosis severity in postmenopausal women than in men with CKD.

Additionally, our study revealed a stronger association between the NPAR and the GS in CKD patients aged 65 years or older. Age is a primary risk factor for the occurrence of atherosclerosis [48], and previous research has confirmed the age-dependent inflammatory characteristics of coronary artery plaques. In elderly patients, coronary plaques exhibit increased CD3^+^ T-cell infiltration and increased myeloperoxidase (MPO) production, indicating a plaque phenotype that promotes atherogenesis and inflammation [49]. Age is also a predictive factor for CKD and is often associated with elevated blood creatinine levels in older patients [50]. Moreover, increasing evidence suggests that chronic low-grade inflammation is one of the mechanisms underlying the aging process, and inflammation has been implicated in most typical age-related chronic diseases [51]. Our results were consistent with these previous results and provided further evidence for the role of age-related inflammation in CKD patients.

Inflammatory markers, which are noninvasive indicators, have the potential to predict the severity of coronary artery lesions, thereby offering novel opportunities for the early identification and treatment of CAD in patients with concurrent CKD. Reducing inflammation has been shown to be effective in treating atherosclerosis and reducing cardiovascular events [52]. Thus, our study demonstrated that the novel NPAR, which is straightforward to calculate, has the potential to serve as a noninvasive indicator to predict the degree of coronary atherosclerosis, thus providing a reference for the selection of CKD patients who should undergo CAG.

There are a few limitations of this study. First, this was a retrospective study, and the causal relationship between the NPAR and the severity of coronary artery disease could not be determined. Second, our study did not consider treatment modalities, which may affect the NPAR or the severity of coronary atherosclerosis. Third, this was a cross-sectional, observational and single-center study, and we did not have long-term follow-up records to evaluate the effect of the NPAR on the prognosis of patients.

## Conclusion

In conclusion, our study revealed that the NPAR is independently associated with the severity of coronary atherosclerosis in CKD patients, especially in women and elderly patients. Higher NPARs are associated with increased rates of complex CAD and increased need for revascularization therapy. The NPAR might serve as a valuable noninvasive biomarker for predicting the severity of coronary atherosclerosis in CKD patients.

## Data Availability

The datasets that were generated and analyzed during the current study are available from the corresponding author upon reasonable request.

## Abbreviations

NPAR: Neutrophil percentage-to-albumin ratio
CKD: Chronic kidney disease
CAD: Coronary artery disease
GS: Gensini score
ROC: Receiver operating characteristic
AUC: Area under the curve
eGFR: Estimated glomerular filtration rate
DM: Diabetes mellitus
TC: Total cholesterol
TG: Triglyceride
HDL‐C: High‐density lipoprotein cholesterol
LDL‐C: Low‐density lipoprotein cholesterol
CAG: Coronary angiography
WBCs: White blood cells
Hb: Hemoglobin
BUN: Blood urea nitrogen
SCr: Serum creatinine
UA: Uric acid
TSH: Thyroid-stimulating hormone
FT3: Free triiodothyronine
FT4: Free thyroxine
LMCA: Left main coronary artery stenosis
TVD: Triple-vessel disease
CTO: Chronic total occlusion
